# Supplementary material for: Comparison of visual quality after wavefront-guided LASIK in patients with different levels of preoperative total ocular higher-order aberrations: a retrospective study
Source: PeerJ. 2024 Aug 21;12:e17940. doi: 10.7717/peerj.17940 (PMC11344536; doi:10.7717/peerj.17940)
Supplement: Supplemental Information 2 [file peerj-12-17940-s002.doc]

STROBE Statement—checklist of items that should be included in reports of observational studies

|  | Item No | Recommendation | **Reported on Page**  **Number/Line**  **Number** |
| --- | --- | --- | --- |
| **Title and abstract** | 1 | Comparison of visual quality after wavefront-guided LASIK in patients with different levels of preoperative total ocular high-order aberrations | 1-3 |
|  |  | **Purpose:** To compare the visual quality after wavefront-guided femtosecond LASIK (WFG FS-LASIK) in patients with different levels of preoperative total ocular high-order aberrations.  **Methods:** This study included 112 right eyes of 112 patients who previously underwent WFG FS-LASIK for correcting myopia and myopic astigmatism. The patients were divided into two groups based on the mean values of preoperative total ocular HOAs (0.30 ± 0.09 µm): HOA ≤ 0.3 and > 0.3 groups. The visual acuity, manifest refraction, corneal Strehl ratio (SR), root mean square (RMS) of corneal and ocular aberrations, and area under the log contrast sensitivity function (AULCSF) of both groups were compared preoperatively and at 1, 3, 6, and 12 months postoperatively.  **Results:** The induced ocular HOAs and coma (Δ = 1mo − Preop) were significantly lower in the HOAs > 0.3 group than in the HOAs ≤ 0.3 group (ΔHOAs: 0.39 ± 0.19 vs. 0.29 ± 0.18 μm, *t* = 2.797, *P* = 0.006; Δcoma: 0.30 ± 0.19 vs. 0.20 ± 0.21 μm, *t* = 2.542, *P* = 0.012). In the HOAs > 0.3 group, ΔHOAs were negatively correlated with the preoperative ocular HOAs (*r* = −0.315, *P* = 0.019). In the HOAs ≤ 0.3 group, the regression equation for Δ HOAs = 0.098 + 0.053\|SE\| (*F* = 21.756, *P* < 0.001). In the HOAs > 0.3 group, the regression equation for ΔHOAs = 0.534 − 1.081HOAs + 0.038\|Sphere\| (*F* = 7.954, *P* = 0.001). The postoperative uncorrected distance visual acuity, spherical equivalent, corneal aberrations, SR and AULCSF of both groups were similar (all *P* > 0.05). Furthermore, the ocular aberrations were not significantly different between both groups at 3, 6, and 12 months postoperatively (all *P* > 0.05). In addition, compared with the preoperative period, the AULCSF of both groups were significantly increased in the postoperative period (all *P* < 0.05).  **Conclusions:** The induced ocular HOAs and coma in HOAs > 0.3 group were lower. Both groups achieved equivalent and excellent visual quality after WFG FS-LASIK. | 19-44 |
| Introduction | | |  |
|  | 2 | Evaluating the visual quality after WFG FS-LASIK in patients with different levels of preoperative total ocular high-order aberrationswill will help clinicians and patients choose appropriate refractive surgery to correct myopia and myopic astigmatism. | 66-69 |
| Objectives | 3 | To compare the visual quality after wavefront-guided femtosecond LASIK (WFG FS-LASIK) in patients with different levels of preoperative total ocular high-order aberrations. | 19-21 |
| Methods | | |  |
| Study design | 4 | Retrospective analysis | 73 |
| Setting | 5 | We evaluated 112 right eyes from 112 patients who underwent WFG FS-LASIK at Chongqing Medal Eye Institute between June 2018 and October 2020. | 73-75 |
| Participants | 6 | The inclusion criteria were as follows: participants aged >18 years, corrected distance visual acuity (CDVA) of 20/20 or better, and stable refraction for >1 year. Patients who were wearing rigid contact lenses were instructed to stop wearing them at least 4 weeks preoperatively, whereas those who were wearing soft contact lenses were instructed to stop wearing them within the previous 2 weeks. | 75-80 |
|  |  |  |  |
| Variables | 7 | The visual acuity, manifest refraction, corneal Strehl ratio (SR), root mean square (RMS) of corneal and ocular aberrations, and area under the log contrast sensitivity function (AULCSF) of both groups were compared preoperatively and at 1, 3, 6, and 12 months postoperatively. | 25-29 |
| Data sources/ measurement | 8* | Ophthalmologic examinations, including logMAR of uncorrected distance visual acuity (UDVA), logMAR of CDVA, slit-lamp microscopic examination of the anterior segments, indirect retinoscopy of the posterior segments, refraction measurements with and without cycloplegia, intraocular pressure (IOP) measurements using a noncontact tonometer (AT555, Rerchert Inc., USA), corneal curvature (ACCUREF-K9001, SHIN-NIPPON Inc., Japan), corneal thickness using an ultrasound pachymeter (300AP＋, SONOMED Inc., USA), corneal topography (Sirius, CSO Inc., Italy), wavefront aberrations (Wavescan Vision 3.68, VISX Inc., USA), and contrast sensitivity (CS) (CSV-1000E, VectorVision Inc., USA), were performed preoperatively and at 1, 3, 6, and 12 months postoperatively.  Corneal tomography (Sirius, CSO Inc.) was performed to identify corneal aberrations in a 6-mm zone. A skilled technician performed at least triplicate measurements on the same eye. The following parameters were analyzed and recorded: Strehl ratio (SR), root mean square (RMS) of total corneal aberrations (TCAs), astigmatism, coma, trefoil, spherical aberrations, and HOAs.  Based on the principle of the Hartmann–Shack wavefront sensor technique, the WaveScan Wavefront aberrometer (Wavescan Vision 3.68, VISX Inc.) was used to measure ocular wavefront aberrations. A skilled technician performed at least triplicate measurements on the same eye with a 6.0 mm pupil diameter. The following parameters were analyzed and recorded: RMS of total aberrations, defocus, astigmatism, coma, trefoil, spherical aberrations, and HOAs.  Monocular best-corrected distance CS was evaluated under scotopic (3 cd/m^2^) condition, photopic (85 cd/m^2^) with and without glare conditions at four spatial frequencies (3, 6, 12, and 18 cycles per degree, c/d). The CS log values at each spatial frequency were recorded, and the area under the log contrast sensitivity function (AULCSF) was calculated for data analysis. | 88-113 |
| Bias | 9 | Patients with diabetes mellitus or autoimmune diseases; those with a history of ocular surgery, trauma, or ocular diseases other than myopia or astigmatism; and those who were nursing or pregnant were not included. | 80-83 |
| Study size | 10 | This study included 112 right eyes of 112 patients who previously underwent WFG FS-LASIK for correcting myopia and myopic astigmatism. | 22-23 |
| Quantitative variables | 11 | As several studies have suggested that the wavefront-guided technology may be more appropriate for patients who have preoperative RMS of ocular HOAs >0.3 μm. Moreover, the mean value of the preoperative RMS of ocular HOAs in our study was 0.30 ± 0.09 µm (range 0.10 – 0.63 µm). Therefore, the patients were divided into two groups: HOAs ≤ 0.3 (57 eyes) and HOAs > 0.3 (55 eyes) groups. The visual acuity, manifest refraction, corneal Strehl ratio (SR), root mean square (RMS) of corneal and ocular aberrations, and area under the log contrast sensitivity function (AULCSF) of both groups were compared preoperatively and at 1, 3, 6, and 12 months postoperatively. | 143-148, 25-29 |
| Statistical methods | 12 | SPSS software (version 26.0; IBM Inc., USA) was used to perform statistical analysis. Data normality was confirmed using the Kolmogorov–Smirnov test. Normally distributed data were represented as mean ± standard deviation. The independent samples t-test and repeated measures analysis of variance were used to analyze the data consistent with normal distribution and homogeneity of variance. Pearson’s correlation and stepwise multiple linear regression analyses were performed to analyze the possible factors affecting ocular HOA induction. χ^2^ analysis was performed to compare the proportions. In case the data were not normally distributed, the Wilcoxon Mann–Whitney test was performed. A *P*-value of <0.05 was considered statistically significant. | 131-140 |

Continued on next page

| Results | | |
| --- | --- | --- |
| Participants | 13* | 1. This study included 112 right eyes of 112 patients with complete clinical data before and 1, 3, 6, and 12 months after surgery. |
|  |  | (b) The patients were divided into two groups based on the mean values of preoperative total ocular HOAs (0.30 ± 0.09 µm): HOAs ≤ 0.3 (57 eyes) and HOAs > 0.3 (55 eyes) groups. |
|  |  |  |
| Descriptive data | 14* | 1. The preoperative age, gender, sphere, cylinder, spherical equivalent, uncorrected distance visual acuity, corrected distance visual acuity, central corneal thickness, non-contact tonometer, pupil diameter, optical zone of both groups were similar (all *P* > 0.05, Table 1). |
|  |  | 1. Preoperatively, the HOAs > 0.3 group had higher RMS of corneal HOAs, trefoil aberrations, RMS of ocular HOAs, coma, trefoil, and spherical aberrations than the HOAs ≤ 0.3 group (all *P* < 0.05). |
|  |  |  |
| Outcome data | 15* | The postoperative uncorrected distance visual acuity (−0.12 ± 0.06 vs. −0.12 ± 0.06, LogMAR), spherical equivalent (0.51 ± 0.39 vs. 0.64 ± 0.51 D), the mean efficacy and safety index (1.01 ± 0.01 vs. 1.00 ± 0.01, 1.01 ± 0.01 vs. 1.01 ± 0.01, respectively) , corneal aberrations (Table 2), SR (0.16 ± 0.04 vs. 0.15 ± 0.04) and AULCSF of both groups were similar (all *P* > 0.05). The ocular aberrations were not significantly different between both groups at 3, 6, and 12 months postoperatively (all *P* > 0.05). The induced ocular HOAs and coma (Δ = 1mo − Preop) were lower in the HOAs > 0.3 group than in the HOAs ≤ 0.3 group (ΔHOAs: 0.39 ± 0.19 vs. 0.29 ± 0.18 μm, *t* = 2.797, *P* = 0.006; Δcoma: 0.30 ± 0.19 vs. 0.20 ± 0.21 μm, *t* = 2.542, *P* = 0.012). In the HOAs > 0.3 group, ΔHOAs were negatively correlated with the preoperative ocular HOAs (*r* = −0.315, *P* = 0.019). In the HOAs ≤ 0.3 group, the regression equation for Δ HOAs = 0.098 + 0.053|SE| (*F* = 21.756, *P* < 0.001). In the HOAs > 0.3 group, the regression equation for ΔHOAs = 0.534 − 1.081HOAs + 0.038|Sphere| (*F* = 7.954, *P* = 0.001). |
|  |  |  |
|  |  |  |
| Main results | 16 | The induced ocular HOAs and coma in HOAs > 0.3 group were lower. The postoperative uncorrected distance visual acuity, spherical equivalent, corneal aberrations, SR and AULCSF of both groups were similar (all *P* > 0.05). Furthermore, the ocular aberrations were not significantly different between both groups at 3, 6, and 12 months postoperatively (all *P* > 0.05). |
|  |  |  |
|  |  |  |
| Other analyses | 17 | Compared with the preoperative period, the AULCSF of both groups were significantly increased in the postoperative period (all *P* < 0.05). |
| Discussion | | |
| Key results | 18 | The induced ocular HOAs and coma in HOAs > 0.3 group were lower. Both groups achieved equivalent and excellent visual quality after WFG FS-LASIK. |
| Limitations | 19 | The limitation of the present study are that it was a retrospective study. Due to the limited number of participants included in the study, we refrained from conducting a grouping analysis based on different levels of myopia. |
| Interpretation | 20 | The induced ocular HOAs and coma (Δ = 1mo − Preop) were lower in the HOAs > 0.3 group than in the HOAs ≤ 0.3 group (ΔHOAs: 0.39 ± 0.19 vs. 0.29 ± 0.18 μm, *t* = 2.797, *P* = 0.006; Δcoma: 0.30 ± 0.19 vs. 0.20 ± 0.21 μm, *t* = 2.542, *P* = 0.012). Pearson’s correlation analysis revealed that ΔHOAs are positively correlated with the preoperative RMS of TOAs, |Sphere|, and |SE|. In the present study, although the difference in preoperative SE was not statistically significant between both groups, it exhibited a slightly increased trend in the HOAs ≤ 0.3 group (−5.09 ± 1.99 and −4.52 ± 1.8 D for the HOAs ≤ 0.3 and HOAs > 0.3 groups, *P* = 0.121).This may be one of the reasons why the HOAs ≤ 0.3 group has more induced HOAs than the HOAs > 0.3 group.  We also observed that preoperative HOAs affected postoperative ocular ΔHOAs. Pearson’s correlation and multiple stepwise regression analyses revealed that ocular ΔHOAs are negatively correlated with the preoperative values of ocular HOAs in the HOAs > 0.3 group (*r* = −0.315, *P* = 0.019). However, this correlation was not observed in the HOAs < 0.3 group (Table 4). Previous studies have reported that the higher the preoperative ocular HOAs, the lower the ΔHOAs. Therefore, some researchers believe most patients with a preoperative RMS HOA of <0.30 μm do not need to undergo WFG LASIK and that wavefront-guided technology provides the greatest benefit for patients with larger preoperative HOA values.  Although the ocular ΔHOA and Δcoma values were lower in the HOAs > 0.3 group than in the HOAs ≤ 0.3 group, the difference in ocular and corneal aberrations was not statistically significant between both groups during follow-up after WFG FS-LASIK (Table 2, 3). This indicates that postoperative corneal and ocular aberrations are consistent, regardless of whether the proportion of preoperative ocular HOAs is high or low. In contrast, Zhang et al. have reported a significant negative correlation between induced changes in ocular HOAs and their preoperative values in both the WFG LASIK and conventional LASIK groups (*r* = −0.577, *P* < 0.001; *r* = −0.443, *P* < 0.001, respectively). ^21^ This finding indicates that ocular ΔHOAs are affected by preoperative HOAs either after WFG LASIK or conventional LASIK.  In the present study, the AULCSF values of the two groups were significantly improved at 1 month postoperatively (Figure 3). No significant differences were observed in the postoperative CSF values at all AULCSF values between both groups (Table 5); this indicates that the visual quality after WFG FS-LASIK was significantly improved in both groups, regardless of whether the RMS of preoperative total ocular HOAs is high or low. |
| Generalisability | 21 | In this retrospective case-control study, the right eye of post-WFG-LASIK patients was included to minimize confounding factors associated with both eyes as subjects. The clinical data of these patients were complete to avoid any potential bias caused by missing data. The grouping in this study was based on a sound rationale. A comprehensive analysis was conducted encompassing visual acuity, manifest refraction, corneal Strehl ratio, root mean square of corneal and ocular aberrations, as well as contrast sensitivity function. Appropriate statistical methods were employed throughout the analysis process. The results obtained are representative. |
| Other information | | |
| Funding | 22 | This work was funded by the Key Program of Chongqing Natural Science Foundation. China (No. cstc2021ycjh-bgzxm0064). |

*Give information separately for cases and controls in case-control studies and, if applicable, for exposed and unexposed groups in cohort and cross-sectional studies.

**Note:** An Explanation and Elaboration article discusses each checklist item and gives methodological background and published examples of transparent reporting. The STROBE checklist is best used in conjunction with this article (freely available on the Web sites of PLoS Medicine at http://www.plosmedicine.org/, Annals of Internal Medicine at http://www.annals.org/, and Epidemiology at http://www.epidem.com/). Information on the STROBE Initiative is available at www.strobe-statement.org.
